# Supplementary figures and images for: Oncolytic Efficacy of a Recombinant Vaccinia Virus Strain Expressing Bacterial Flagellin in Solid Tumor Models
Source: Viruses. 2023 Mar 24;15(4):828. doi: 10.3390/v15040828 (PMC10142208; doi:10.3390/v15040828)

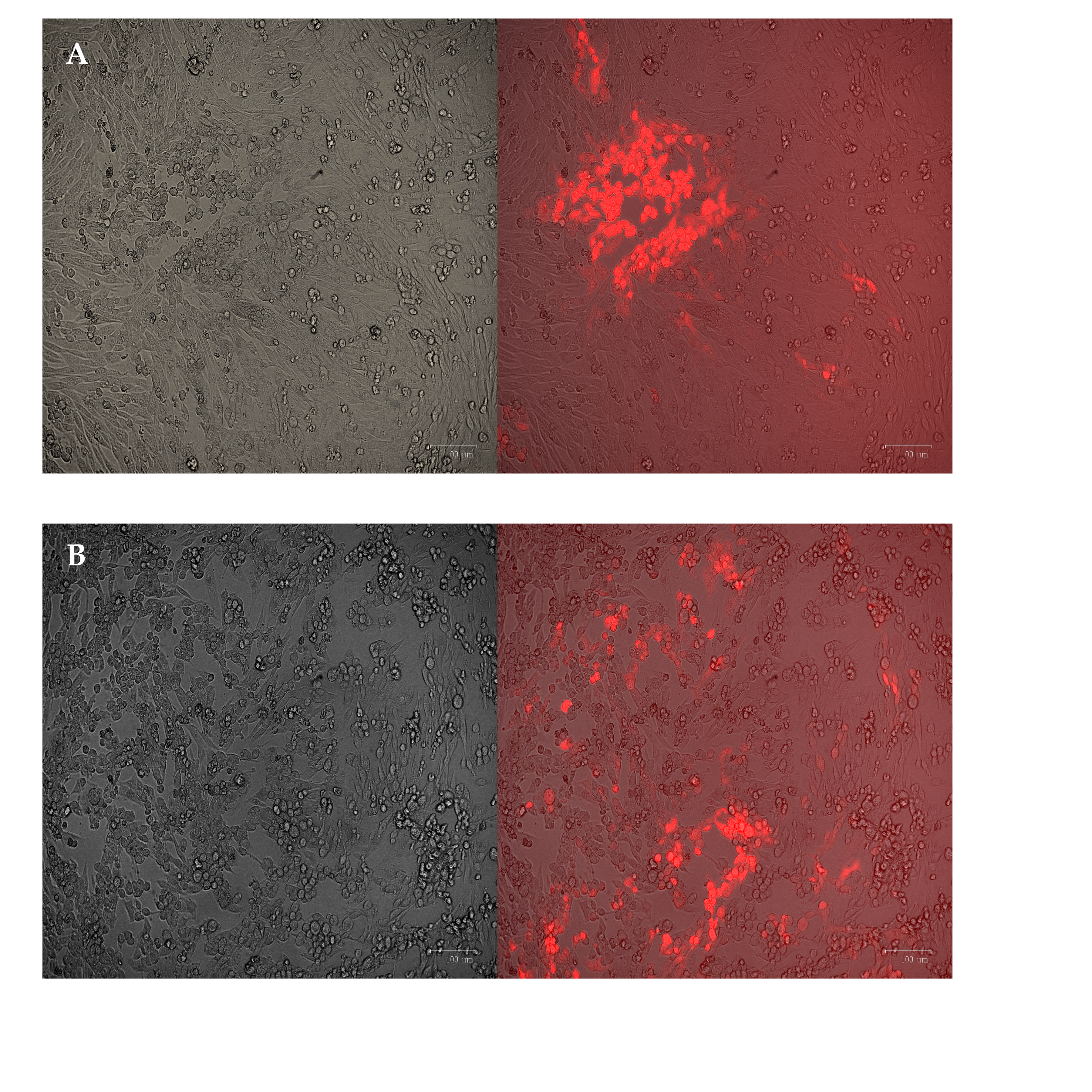

Supplement: Supplementary file 1 [file viruses-15-00828-s001.zip › FIGURE-SUPPLEMENTARY 1- infected cells by LIVP-RFP(A), LIVP-Fluc-RFP (B).tif]

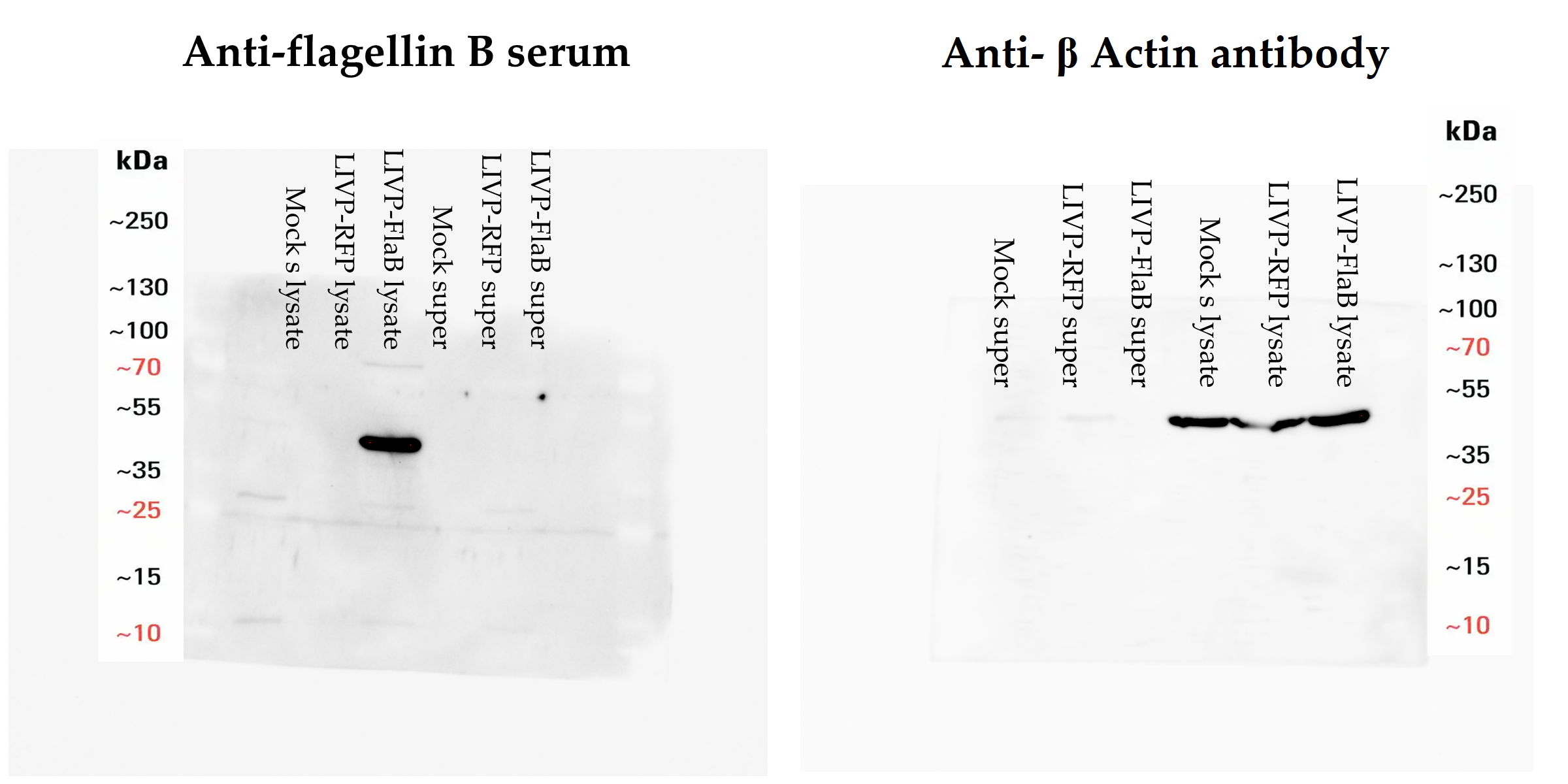

Supplement: Supplementary file 1 [file viruses-15-00828-s001.zip › FIGURE-SUPPLEMENTARY 2- uncropped blot.tif]

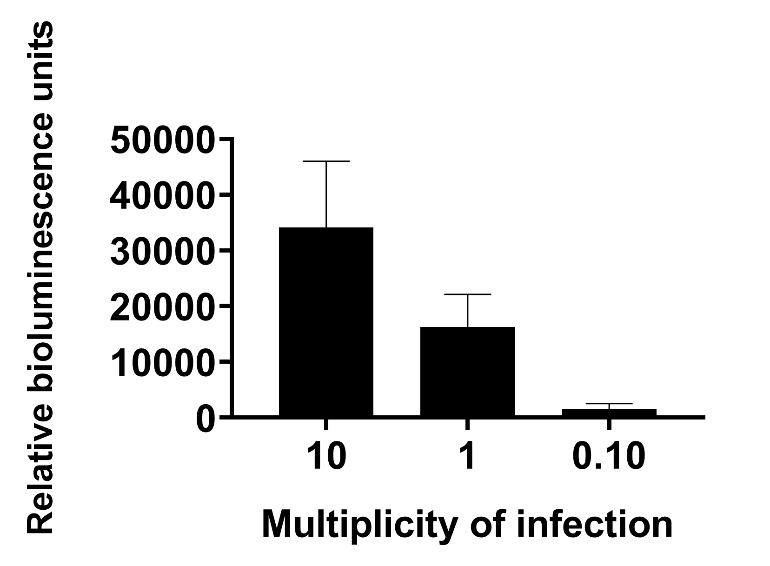

Supplement: Supplementary file 1 [file viruses-15-00828-s001.zip › FIGURE-SUPPLEMENTARY 3- bioluminescence.tif]

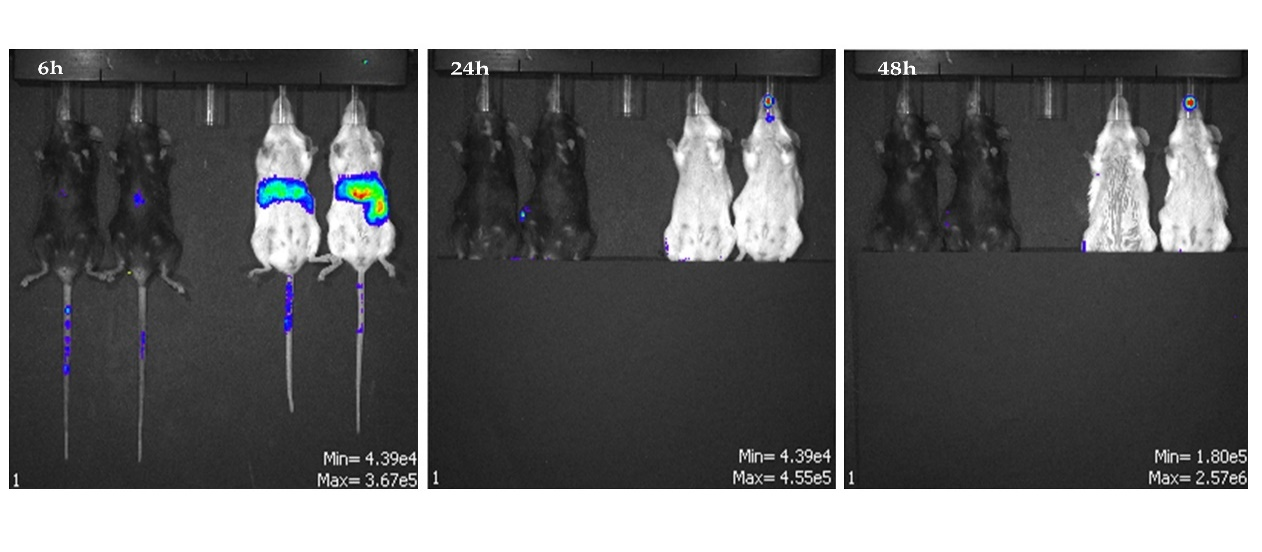

Supplement: Supplementary file 1 [file viruses-15-00828-s001.zip › FIGURE-SUPPLEMENTARY 4- IVIS.tif]

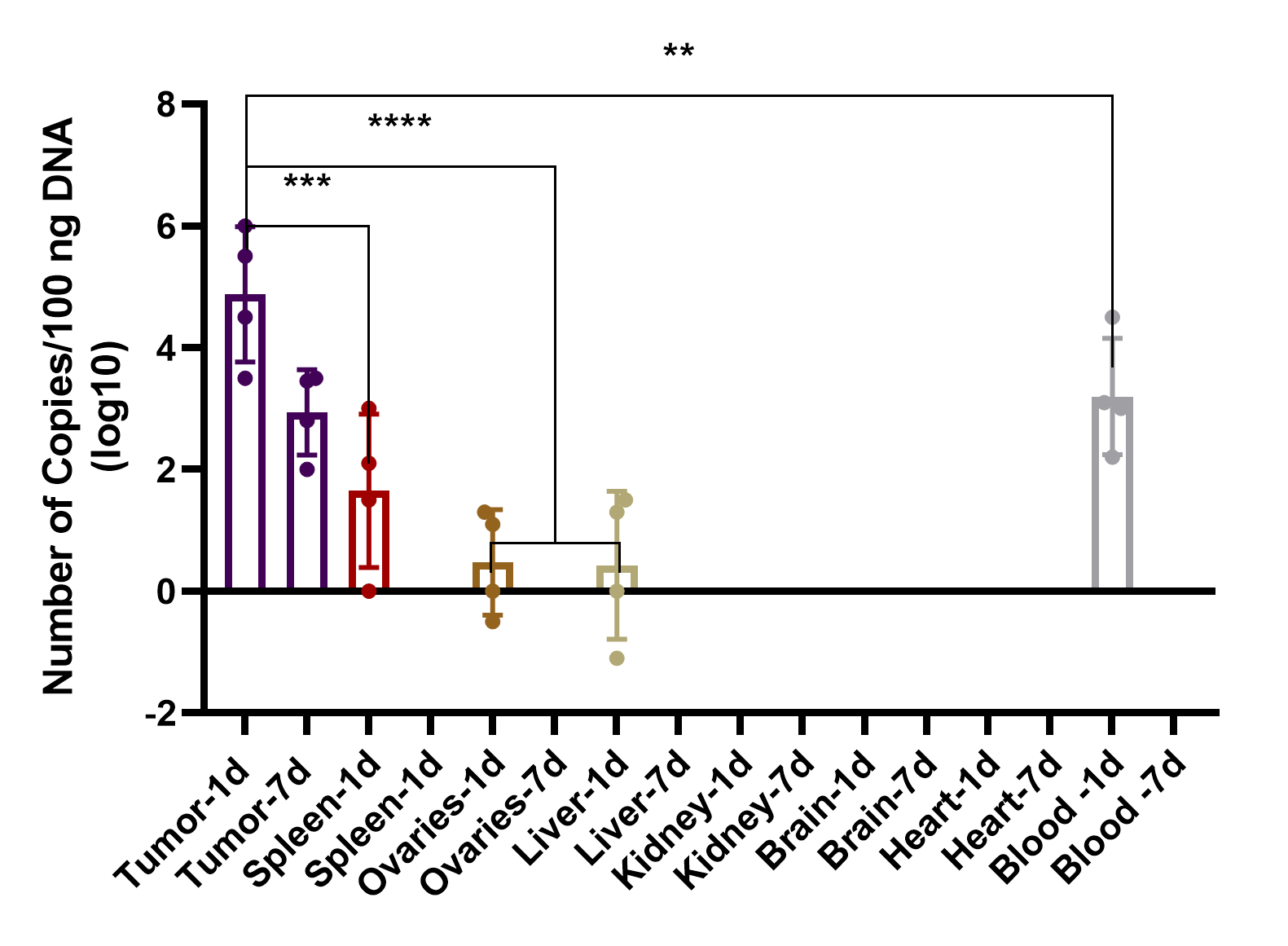

Supplement: Supplementary file 1 [file viruses-15-00828-s001.zip › FIGURE-SUPPLEMENTARY 5- qPCR.tif]

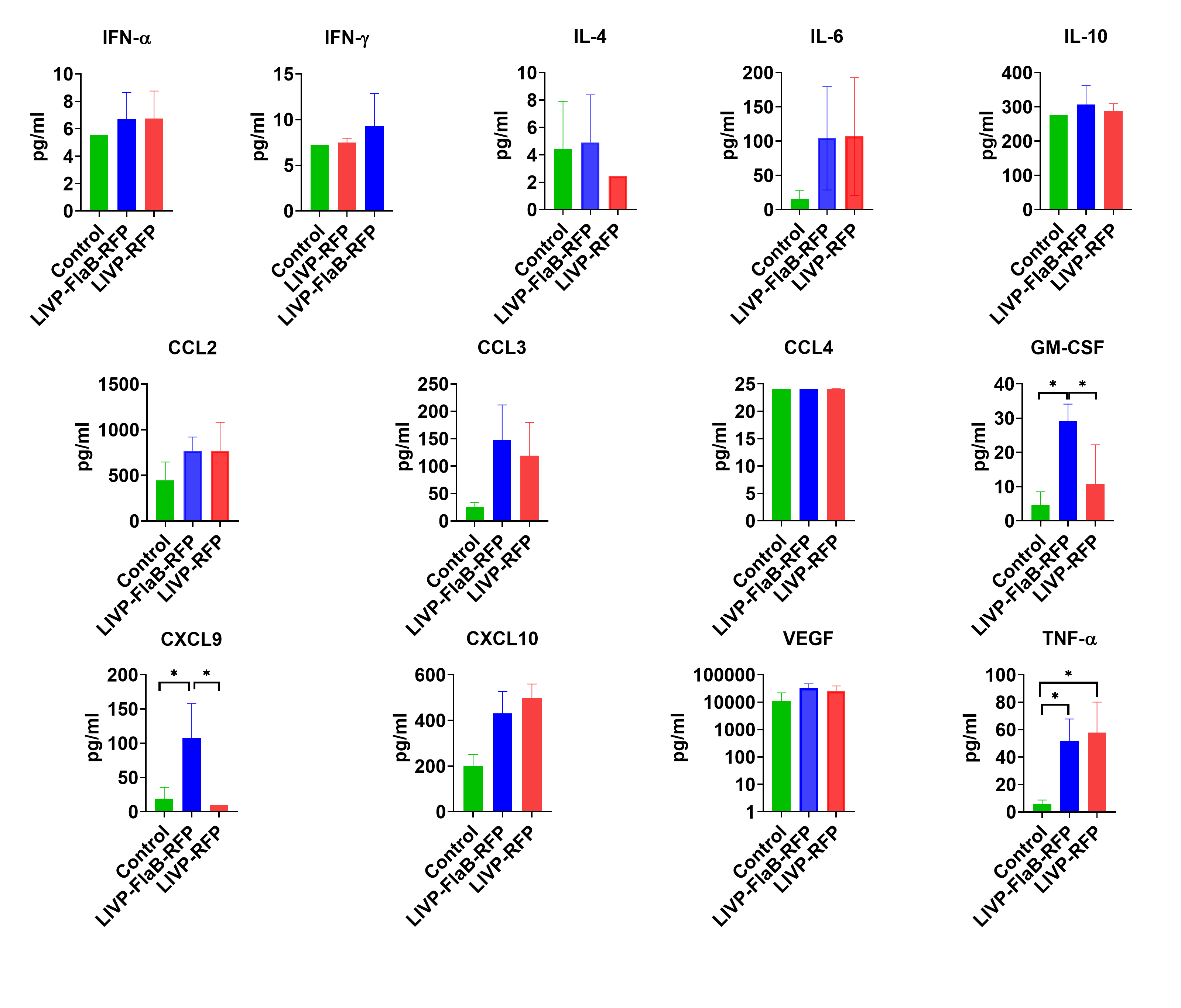

Supplement: Supplementary file 1 [file viruses-15-00828-s001.zip › FIGURE-SUPPLEMENTARY 6- tumor infiltrated fluids cytokine analysis.tif]

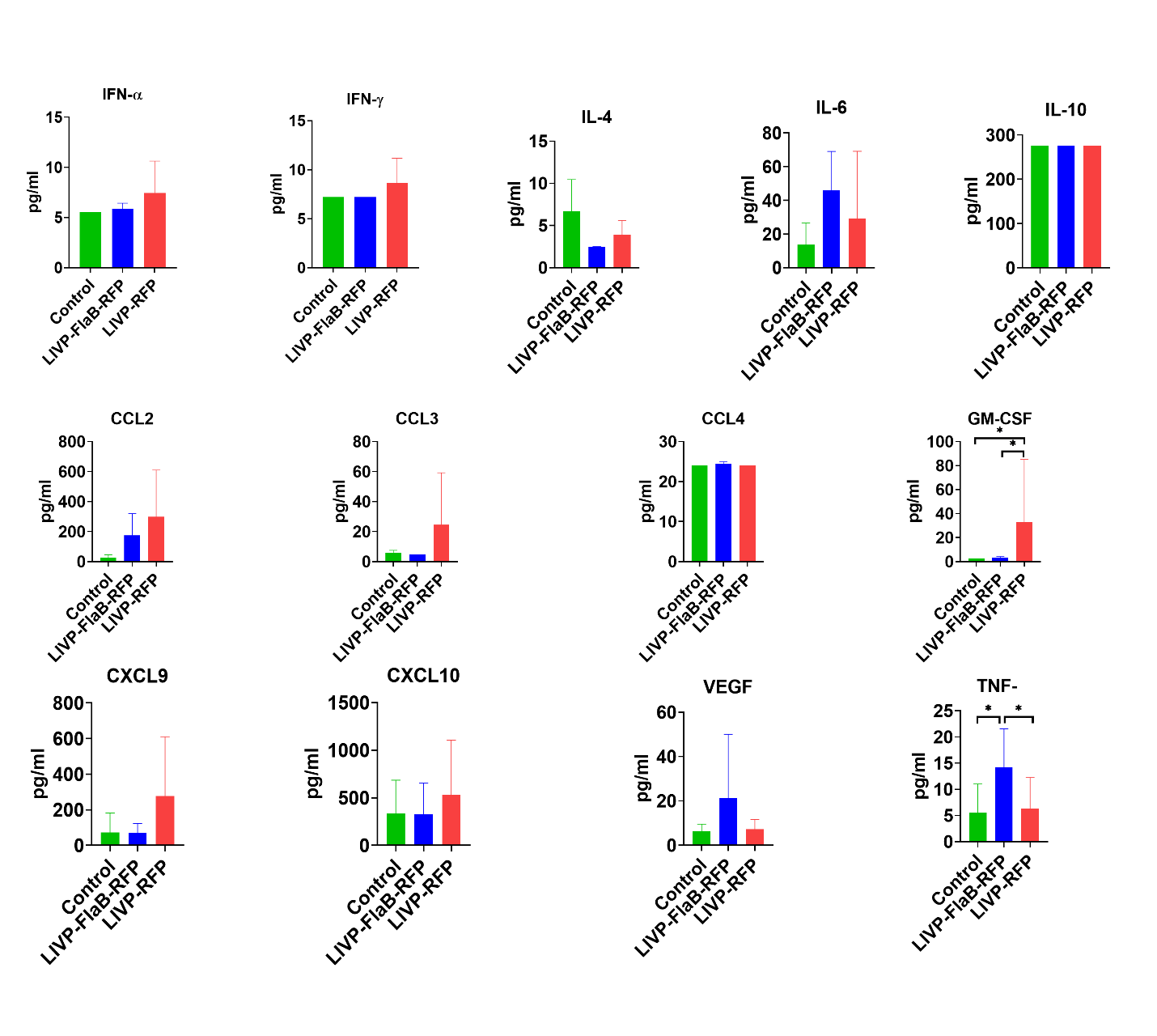

Supplement: Supplementary file 1 [file viruses-15-00828-s001.zip › FIGURE-SUPPLEMENTARY 7- serum cytokine analysis.tif]

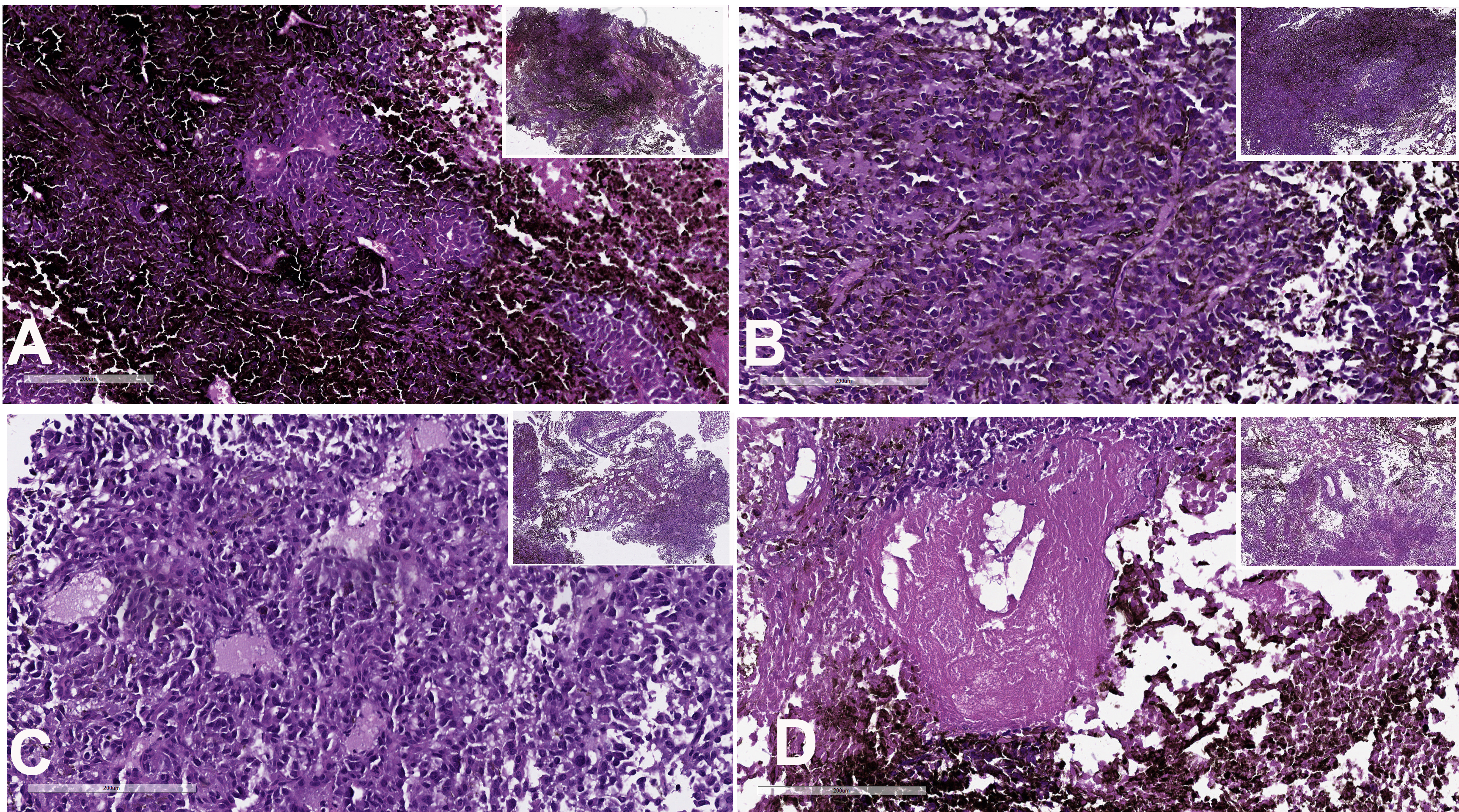

Supplement: Supplementary file 1 [file viruses-15-00828-s001.zip › FIGURE-SUPPLEMENTARY 8- Histology.tif]
